# Anatomy of the Clitoris: Revision and Clarifications about the Anatomical Terms for the Clitoris Proposed (without Scientific Bases) by Helen O'Connell, Emmanuele Jannini, and Odile Buisson

**DOI:** 10.5402/2011/261464

**Published:** 2011-09-15

**Authors:** Vincenzo Puppo

**Affiliations:** Centro Italiano di Sessuologia (CIS), Via Regnoli 74, 40138 Bologna, Italy

## Abstract

The anatomy of the clitoris is described in human anatomy textbooks. Some researchers have proposal and divulged a new anatomical terminology for the clitoris. This paper is a revision of the anatomical terms proposed by Helen O'Connell, Emmanuele Jannini, and Odile Buisson. Gynecologists, sexual medicine experts, and sexologists should spread certainties for all women, not hypotheses or personal opinions, they should use scientific terminology: clitoral/vaginal/uterine orgasm, G/A/C/U spot orgasm, and female ejaculation, are terms that should not be used by sexologists, women, and mass media. Clitoral bulbs, clitoral or clitoris-urethrovaginal complex, urethrovaginal space, periurethral glans, Halban's fascia erogenous zone, vaginal anterior fornix erogenous zone, genitosensory component of the vagus nerve, and G-spot, are terms used by some sexologists, but they are not accepted or shared by experts in human anatomy. Sexologists should define have sex, make love, the situation in which the orgasm happens in both partners with or without a vaginal intercourse.

## 1. Introduction

Findings from the disciplines of embryology, anatomy, and physiology of the clitoris should form the basis of the discourse about the biological basis of the female orgasm. The anatomy of the clitoris is described in human anatomy textbooks, but often it is neglected by sexological textbooks, for this reason some researchers have proposal and divulged a new anatomical terminology for the clitoris. This review is a revision of the anatomical terms proposed by Helen O'Connell, Emmanuele Jannini, and Odile Buisson.

## 2. Methods

This is a revision of the Helen O'Connell and Emmanuele Jannini articles published by Journal of Sexual Medicine and of Odile Buisson's article published by Gynécologie Obstétrique Fertilité.

O'Connell et al. in The Anatomy of the Distal Vagina: Towards Unity (Journal of Sexual Medicine, 2008) wrote: “The aim of this presentation is to provide a comprehensive overview of anatomy of the distal vagina.” “This would aid communication between clinicians, researchers, and the nonclinician regarding this anatomy.” “The other components are the walls of the vagina and its associated exocrine glands,” “which will focus instead on the distal vagina, the site of the female sexual response and the area where confusion in terminology most exists” [1].

O'Connell et al. fail to describe the “anatomy of the distal vagina”: for instance in her article nothing is written about the size of the “distal vagina” and of its gross and microscopic anatomical structure. Moreover, the term “distal vagina” is not used in human anatomy and there are no exocrine glands in the walls of the vagina; the focus of the female sexual response is not the “distal vagina,” but the clitoris (with its glans: the female primary erogenous zone, which covers the distal part of the corpora cavernosa of the clitoris from which it is independent). There is general consensus about female sexual anatomy and the standard terminology employed in this respect, such as the anatomical terms used to describe the components of the vulva in all human anatomy textbooks [2–7].

O'Connell et al. wrote: “There is no uniformity to the vagina, the distal vagina having distinctly different properties to the proximal vagina, reflecting their different developmental origins from the urogenital sinus and Mullerian duct [3]” [1].

Fliegner, quoted by O'Connell et al. [8], in 1994 writes [9]: “A review of the embryology of vaginal epithelium suggests that it is entirely of urogenital sinus origin.”

The vagina and cervix develop without the involvement of the Müllerians ducts; only just the body of the uterus and uterine tubas are formed by the Müllerian ducts, and in males the prostatic utricle is the homologue of the female vagina [2, 5, 6, 10].

“There is also substantial variation in the structure of the walls. For example, the lateral walls are very different from the posterior vaginal wall” [1].

For this statement, in O'Connell et al.'s article, there are no references and the authors do not clarify if they relate to the gross or microscopic anatomy and they do not write the differences between lateral and posterior walls of the vagina.

“The distal vagina is a structure that is so interrelated with the clitoris that it is a matter of some debate whether the two are truly separate structures.” “Deep to the vaginal wall mucosa laterally lies only the clitoris.” [1].

**Figure 1:**
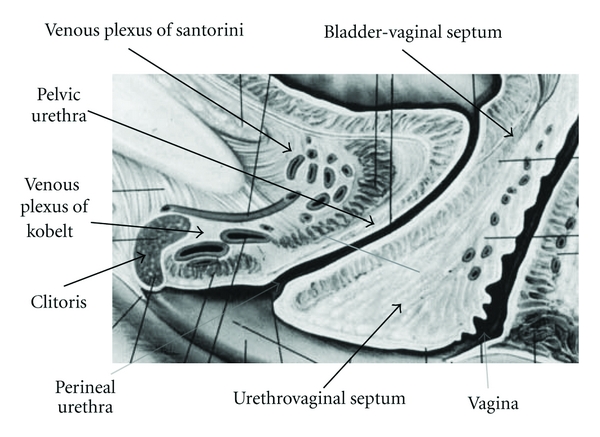
The clitoris, the pelvic and perineal urethra, and the vagina. Modified from [6].

The vagina has no anatomical relation with the clitoris (Figure 1) [2–7].

“The urethra is separated from the distal vagina by a discrete white layer referred to as the periurethral fascia” [1].

The anterior vaginal wall is separated from the posterior urethral wall by the urethrovaginal septum (its thickness is 10–12 mm) [2–7].

“Although the literature written for lay readers implies that the urethra is erectile in nature or is surrounded as a tube by vessels, this idea is not supported by anatomical studies” [1].

The corpus spongiosum of the female urethra is reported in anatomy textbooks [5–7] and in anatomical studies: Grafenberg, in 1950, writes “Analogous to the male urethra, the female urethra also seems to be surrounded by erectile tissues like the corpora cavernosa” [11], Yang et al. in 2006, write “The spongy tissue surrounding the urethral lumen is composed of smooth muscle fibres, with multiple small vessels (Figure 10). Some have termed this the corpus spongiosum, as in the male” [12].

“The labiae, like the clitoris, are derived embryologically from the undifferentiated phallus” [1].

Only the corpora cavernosa of the clitoris and the glans are formed from the phallus. Labia minora, vestibule of the vagina, and vestibular bulbs are formed from the pelvic and phallic parts of the urogenital sinus and from the urogenital folds [2, 5, 6, 13–15].

O'Connell et al. wrote “Clitoral bulbs” [1]. Clitoral bulbs is an incorrect term from an embryological and anatomical viewpoint, in fact the bulbs do not develop from the phallus, and they do not belong to the clitoris: “clitoral bulbs” is not a term used in human anatomy, the correct term is the vestibular bulbs [2–7, 13–17].

“The urethral orifice and distal urethra are surrounded by the erectile tissue of the clitoral bulbs.” “The urethra is encircled by the clitoris to a variable degree” [1].

The external urethral orifice is situated on the vaginal tubercle (i.e., carina urethralis) and it is not surrounded by the erectile tissue of the vestibular bulbs; the female urethra is not encircled by the clitoris, and it is not in related with the crura and body of the clitoris [2–7, 13–15]; the female urethra is only 3-4 cm long and the authors do not clarify the meaning of “distal urethra.”

“The clitoris is composed of the glans, which is its only external manifestation.” “the clitoris itself, in turn, being covered by the vulva” [1].

The clitoris is not “covered by the vulva”: it is a part of the vulva. The whole clitoris (glans, body, roots, or crura) is an external genital organ: the glans and body are visible while the roots are hidden, therefore they are not “internal” [2–7, 13–15].

“The tiny glans clitoris, a component of the clitoris composed primarily of large nerve trunks and sensory receptors, is often referred to as the clitoris.” [1].

In O'Connell et al.'s article there are no references for this assertion, and in the pictures of the vulva in anatomy textbooks always there is the body of the clitoris [2–7].

“This area was emphasized in the descriptions of Kobelt, and more recently by Arien, Sevely, and Douglas.” [1].

The author “Arien” cited by O'Connell et al. does not exist, and Reference 29 is not correct, the authors are not “Arjen A, Turnhout V, Hage J, Diest P.”, but Van Turnhout AA, Hage JJ, van Diest PJ [16]. Also in Reference 11 of O'Connell et al.'s article there is an error, the article by Yang C. et al., it is not published in “2005,” but in 2006 [12].

“The interrelationship between the clitoris, urethra, and vagina has been studied by various modalities including ultrasound [34], MRI [35]” [1].

The authors quoted in Reference 35 by O'Connell et al. are Georgiadis et al. [18], also this is an incorrect reference, this article does not investigate the interrelation between the clitoris, urethra, and vagina, and MRI was not used, Georgiadis et al. wrote: “The importance of the clitoris for female sexual pleasure is undisputed. However, this is the first account of brain regions involved in the experience of clitoral stimulation … PET was used because it is more robust to motion artifact than fMRI” [18].

“The clitoral complex, composed of the distal vagina, urethra, and clitoris, is the location of female sexual activity, analogous to the penis in men” [1].

This definition has no embryological, anatomical, and physiological support and in the male penis there is no vagina [2–7, 13–16, 19]. Dickinson, in 1949, wrote [7]: “The general homology between the male and female genitalia is too well know.” Laqueur, in 1990, wrote that how Adam, Renaldus Columbus baptized (“amoris dulcedo”) in 1559, what he had found in nature: a female penis [20]. To describe the cluster of erectile tissues (i.e., clitoris, vestibular bulbs and pars intermedia, labia minora, and corpus spongiosum of the female urethra) responsible for female orgasm, the correct anatomical term should be the female penis [13–15].

In Measurement of the Thickness of the Urethrovaginal Space in Women With or Without Vaginal Orgasm: A Response from the Study Authors (Journal of Sexual Medicine, 2008), answering to “Rebuttal” of Professor Vicentini [21], Jannini stated that: “Female sexuality is still a hidden area of medicine, which needs an honest, expert scientific approach in order to move forward” [22]. This is not a very appropriate statement, because there are many scientific mistakes in the article written by him with Gravina et al. [23].

Gravina and Jannini et al. wrote: “Gräfenberg described an erogenous zone located in the anterior vaginal wall and subsequent studies have correlated the focus of female sensitivity with the external urethral sphincter” [23].

G-spot of Ladas, Whipple, and Perry does not correspond to the external urethral sphincter but to the intraurethral glands: Komisaruk et al., in 2006 write “Stimulation of the pelvic nerve may also occur with stimulation of the area of the G-spot (the area of the female prostate gland) and may also account for the reports of orgasm and female ejaculation from the urethra experienced by some women” (and why in this book “the science of orgasm” nothing is written about the clitoris?) [24].

Furthermore the authors also write: “between the thickness of urethrovaginal space, or G-spot,” “clitoris-urethrovaginal complex, also known as the G-spot” [23]: therefore in the same article Gravina and Jannini et al. wrote 3 definitions of G-spot and each one of them is incorrect.

Gravina and Jannini et al. write that they made an echography of the G-Spot [23], but in their article there is no picture that shows a G-spot!

“Female genital anatomy and the physiology of female sexual function have been scientifically neglected in the past” [23].

The female genital anatomy has been described in Human Anatomy textbooks and the female genital physiology has been described for the first time in Dickinson's textbooks in 1949 and subsequently by Masters and Johnson [2–7, 15, 19, 25].

“The urethrovaginal space (where the Halban's fascia runs) seems critical, being constituted of fibroconnective tissue and large numbers of blood vessels, glands, muscular fibers, and nerve endings” [23].

Urethrovaginal space is an incorrect term from a scientific point of view, the anterior vaginal wall is separated from the posterior urethral wall by the urethrovaginal septum [2–6]. In addition Halban's fascia, a layer of dense connective situated in the bladder-vaginal septum, does not correspond to the male corpus spongiosum as some sexologists believe: this assumption has no embryological and anatomical support [5, 6, 26].

“The most interesting finding of our study is the evidence that women who experience vaginal orgasm have an urethrovaginal space thicker than those who do not … The self-reported nature of presence or absence of vaginal orgasm is another strong limitation of our findings … By vaginal orgasm we mean the orgasm experienced after direct stimulation of the anterior vaginal wall by penetration” [23].

One vaginal orgasm “at least once in the past month,” in women that “reported at least two acts of sexual intercourse per week” [23], is not a meaningful difference with women without vaginal orgasm, besides authors did not clarify the measures of this “space” considered as normal, and they do not specify the position of coitus.

“As there is now evidence that the clitoris is related to the distal third of the urethra in the perivaginal space … The close physical proximity of the urethra and the clitoris to the anterior vaginal wall suggests an association between these anatomical structures and sexual function … The presence of pseudocavernous tissue (clitoral bulb) in the anterior vaginal mucosa is a frequent but not universal finding (86%)” [23].

The vagina has not anatomical relation with the clitoris and in the anterior vaginal mucosa there is no “clitoral bulb” [2–7]!

“In fact, the anterior vaginal wall is an active organ, transmitting, during intercourse, the effect of penile thrusting in the vagina to the clitoris, by stretching the two ligaments that insert around its base” [23].

Gravina and Jannini et al. have quoted a “Current Opinion” of 200 words [27]: this statement is not corroborated by any anatomical or physiological evidence.

“However, our data cannot directly demonstrate that the thickness of an anatomical space may generate a mechanism that can be related to the creation of an orgasm … But, in conclusion, the results here presented allow us to speculate that there may be a functional correlation between the thickness of urethrovaginal space, or G-spot, and the ability to experience the vaginal orgasm” [23].

Sexual medicine experts should spread scientific notions and not speculations!

G-spot is not a term used in human anatomy: Grafenberg, in 1950, describes some cases of female and male urethral masturbation and the corpus spongiosum of the female urethra. Grafenberg wrote that the intraurethral glands could only release fluids that are not urine during the orgasm: but he did not report an orgasm of intraurethral glands. There are no ultrasonographic images or anatomical pictures of the G-spot, and the female prostate has no anatomical structure that can cause an orgasm: G-spot does not exist and the hypothetical area named G-spot should not be defined with Grafenberg's name [11, 13, 28].

In The G-spot and Lack of Female Sexual Medicine (Gynécologie Obstétrique Fertilité 2010), Odile Buisson writes: “G-spot was popularized by sexologist Beverly Whipple in 1980 in honor of the gynecologist Ernst Grafenberg”, “the exact anatomy of the clitoris only recently has been recognized”, “the dynamic study of the clitoris urethra-vaginal complex,” “the vaginal penetration causes a close contact between the inner clitoris and the distal anterior vaginal wall” [29].

G-spot is a hypothesis, and there is no anatomical evidence of the vaginal orgasm which was invented by Freud in 1905, without any scientific basis [11, 13, 19, 20, 28, 30].

Clitoris is localized under the urogenital diaphragm, in front the pubic symphysis and in the anterior perineal region and the roots of the clitoris are located in contact with the ischiopubic ramus, covered by the ischiocavernosus muscles: they cannot come into contact with the anterior vaginal wall and the “inner clitoris” does not exist [2–7, 13, 30].

**Figure 2:**
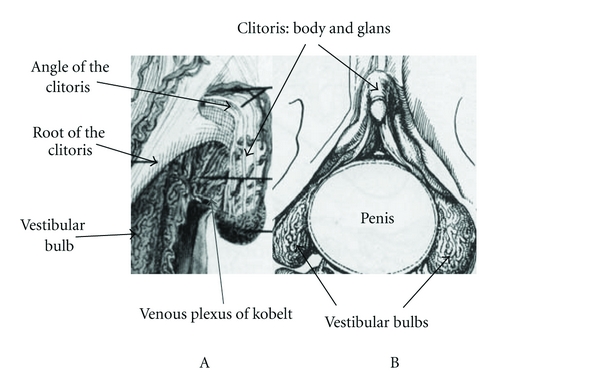
The male penis cannot come in contact with the venous plexus of Kobelt or with the roots of the clitoris. (A) Modified from [6] (B) Modified from [7].

The female perineal urethra, which is located in front of the anterior vaginal wall, is about one centimeter in length and the G-spot is located in the pelvic wall of the urethra, 2-3 cm into the vagina. The male penis cannot come in contact with the venous plexus of Kobelt (situated until the angle of the clitoris) or with the roots of the clitoris (which do not have sensory receptors or erogenous sensitivity) during vaginal intercourse (Figure 2) [2–7, 13, 30].

Buisson stated that the clitoris is composed of two arcs, the first consisting of two corpora cavernosa along the right and left ischiopubic ramus, with a length of 12–15 cm; they join on the summit of the vulva to form a bend 90 degrees forward: the raphe; the raphe ends in the glans clitoris, the visible part of the clitoris. The second arc consists of two bulbs that surround the lateral walls of the vagina [29]. This Buisson's statement is not corroborated by any embryological, anatomical, or physiological evidence: the clitoris is not composed of “two arcs” [2–7, 12, 13].

In the article by O. Buisson there are only hypotheses to explain others that are conclusions without scientific basis (and she does not write how many women would have the G-spot): the clitoral stimulation is important to have an orgasm and the clitoris exists in all women (i.e., 100%!), why not simply stimulate, during intercourse with penetration of the penis, the clitoris with a finger [30]?

## 3. Conclusions

The meaning of words is important in science, but particularly in the female sexuality: gynecologists, sexual medicine experts, and sexologists should spread certainties for all women not hypotheses or personal opinions, they should use correct scientific terminology: clitoral/vaginal/uterine orgasm, G/A/C/U spot orgasm, female ejaculation, are terms that should not be used by sexologists, women, and mass media; clitoral bulbs, clitoral or clitoris-urethrovaginal complex, urethrovaginal space, periurethral glans, Halban's fascia erogenous zone, vaginal anterior fornix erogenous zone, genitosensory component of the vagus nerve, and G-spot, are terms used by some sexologists but they are not accepted or shared by experts in human anatomy [13].

Women have the right to feel sexual pleasure [31]: in all healthy women, orgasm is possible, with effective stimulation [13, 19, 25, 32, 33]. Make sex, make love, do not need necessarily to finish with an intercourse, and the female orgasm can be triggered by various noncoital sex play, that is, foreplay, partner masturbation, and cunnilingus [25]. Sexologists should define have sex, make love, the situation in which the orgasm happens in both partners with or without a vaginal intercourse [32–34].

I hope that this review article will give rise to some fruitful discussions on the topic of female sexuality.
